# Exploring the feasibility of collecting music and wellbeing data to examine intentional listening using a mobile-ESM application

**DOI:** 10.3389/fpsyg.2025.1505929

**Published:** 2025-02-28

**Authors:** Katrina Skewes McFerran, Amanda E. Krause, Margaret S. Osborne

**Affiliations:** ^1^Faculty of Fine Arts and Music, The University of Melbourne, Melbourne, VIC, Australia; ^2^Department of Psychology, James Cook University, Townsville, QLD, Australia; ^3^Melbourne School of Psychological Sciences, The University of Melbourne, Melbourne, VIC, Australia

**Keywords:** everyday music listening, experience sampling methodology (ESM), flourishing, well-being, mobile listening, MuPsych

## Abstract

This brief report describes a small-scale feasibility study investigating the use of mobile Experience Sampling Methodology (ESM) for collecting data on intentionality in music listening for well-being. Sixteen university students used the MuPsych app (Randall and Rickard, 2012) for a 2-week pilot study (resulting in 263 music listening episode responses), with seven participating in semi-structured follow-up interviews. Data was collected at baseline and then triggered by mobile music listening episodes at 0, 5 and 20 min. Baseline measures were of wellbeing; and listening episode data included music choice, purpose, context, and mood. After assigning listeners to languishing, moderate, or flourishing wellbeing categories, differences became apparent in participants’ experiences of listening to music. Several challenges to feasibility were experienced in self-selection and biased reporting by participants as well as technological limitations of data collection techniques. Recommendations for future ESM studies of everyday music listening are offered.

## Introduction

Experience Sampling Methodologies (ESM) collect self-report data in-situ, affording ecological validity and capturing perspectives during the activity rather than relying on retrospective reconstruction (van Berkel et al., 2017). Repeated measurements and comparisons are also enabled. Mobile technologies have enabled detailed ESM studies and have been utilised across a range of fields with increasing popularity (Fritz et al., 2024).

ESM data collection has also been popular in the field of music psychology. When first trialled by Sloboda et al. (2001), eight participants reported their thoughts and feelings when music was playing (44% of instances) with very high levels of compliance and motivation to participate being reported. North et al. (2004) then used a similar method to examine who people were listening with, what they were listening to, when they listened, where they listened, and why they listened. Sending one text message per day for 14 days, they surveyed 346 people, again noting a high level of compliance that enabled robust analysis of contextual information. Shortly after, Juslin et al. (2008) conducted a comprehensive ESM study investigating the prevalence of different musical emotions and how such emotions were related to various factors in the listener, the music, and the situation. This time, data was collected seven times a day for two weeks allowing situational sampling and detailed analysis that illustrated the benefits of understanding people’s subjective experiences of music through repeated sampling *in situ*. These studies established the value and feasibility of ESM for music psychology research.

Since then, technological advancements have created improved opportunities for data collection about music listening. Mobile technologies mean that people can listen to music even more easily and efficiently, so much so that new concerns have now emerged about the dangers of pedestrians distracted by music listening (Simmons et al., 2020). Streaming services mean that any music can be found at a moment’s notice, and this has changed the ways that people select and consume music, raising some concerns (Hesmondhalgh, 2022). Tracking technologies are embedded in the same mobile devices as music, making a wide array of data available about location that again raises security issues (Ribeiro-Navarrete et al., 2021) but also opportunities and emerging interest in combining survey and location data for research purposes (Stier et al., 2020). Technologies are now developing so quickly that it can be challenging to identify what technologies are worth investment.

One tool that has remained in active use in music psychology studies in the MuPsych app (Randall and Rickard, 2012), initially developed and used in Australia (Randall and Rickard, 2017; Randall et al., 2014) and now integrated into a range of studies within the Finnish Centre of Research Excellence in Music, Mind, Body and Brain (Randall et al., 2022; Saarikallio et al., 2019) and others (Ruth et al., 2023). The group have claimed that it enables research that has strong ecological validity and that it is adaptable to a range of research goals (Saarikallio et al., 2020).

Whilst ESM research, to date, has increased understanding of music listening for emotional regulation (e.g., Randall and Rickard, 2012; Saarikallio et al., 2020), the success of deliberate, self-directed, goal focused listening with a wellbeing intention has not been examined *in situ*. However, retrospective data suggests that some people may listen to music with the hope of feeling better, but due to poor mental health conditions may feel worse through mechanisms such as rumination, isolation and intensification (McFerran and Saarikallio, 2014). Therefore, it would be useful within an ESM study paradigm to compare music listening experiences according to self-reported levels of wellbeing which range from languishing to flourishing (to use Keyes’ vocabulary, Keyes, 2002).

In order to undertake future research on the success of intentional music listening to achieve wellbeing goals, we needed to identify the most useful ESM methods. This brief report examines the fidelity of the methods deemed most likely to be suitable by reporting on a trial of the technology, the engagement with requested tasks, and the effectiveness of the proposed measures. Specifically, our feasibility study aimed to answer the following questions:

Research question 1. How practical and engaging is using mobile-ESM to collect data from younger adults on intentionality in music listening and wellbeing?

Research question 2. How effective is collecting data triggered by music listening (i.e., that is collected at 0, 5, and 20 min after music listening begins) for answering questions about the connection between music listening, goal achievement and wellbeing? Specifically, can different patterns of music listening be observed across languishing, moderate and flourishing levels of wellbeing?

## Method

### Design

This brief report describes a feasibility study which examined whether it was practical, acceptable and effective (Bowen et al., 2009) to use a listening-episode-triggered mobile experience sampling methodology to understand the connection between intentional music listening and wellbeing. The study obtained ethics approval through [reference removed] (2023–25,647–43,544-5).

### Participants

A convenience sample of 16 university students (*Mage* = 24.13, *SDage* = 6.05; 56.3% female, 31.3% male, 6.3% non-binary) residing in Australia (*n* = 6) and Singapore (*n* = 9) participated in this feasibility study. Eligibility requirements were being 17+ years of age and listening to music on an Android phone, which was a requirement for downloading the MuPsych app (Randall and Rickard, 2012). Exclusion criteria included being engaged in treatment for serious mental health issues to ensure participants were sampled from the general population. University research participation programs were used to recruit undergraduate psychology students, such that individuals received course credit for their participation.

### Procedure and materials

Participants were asked to download the MuPsych app. Upon doing so, they were asked within the app to consent to participate and complete background information (including demographic questions and standard MuPsych background surveys) (Randall and Rickard, 2012) before commencing episode-triggered data collection. For the next two weeks, the MuPsych app was activated when they began listening to music on their Android phone (e.g., opening Spotify and playing a song). Participants were prompted to answer questions immediately (0 min), and then again at 5 and 20 min into a listening session. Participants were also invited to participate in a follow-up interview after the two-week period.

### MuPsych

We used the standard MuPsych battery of self-report measures (Randall and Rickard, 2012; Randall et al., 2022) for the 0, 5, and 20 min surveys (asking about contextual conditions including mood and arousal [slider scale responses] and reasons for listening [selecting from MuPsych’s standard list of options]). In addition, for this study, we added a single well-being question at each time point, as well as two further questions about success and choice at 5 and 20 min. These items and response scales were as follows:

- Wellbeing: “*Right now, how would you rate your level of wellbeing?,”* −3 “Very poor” to +3 “Excellent.”
- Success: “*Did you achieve your music listening goal?,”* −3 “Not at all” to +3 “Completely”
- Choice: *“How deliberate/specific was your choice of music?,”* −3 “Not at all (I could have listened to anything)” to +3 “Very (I needed to hear this specific music).”

### Wellbeing categorisation

At the beginning of study, participants were asked to complete a background survey to reflect on their level of wellbeing during the previous month using the *Mental Health Continuum Short Form* (MHC-SF; Keyes, 2014). The MHC-SF is a 14-item self-report scale that asks participants to indicate on a Likert scale (0 = *None* to 5 = *Every day*) how often they have experienced aspects of emotional, social and psychological wellbeing. This yields an overall wellbeing score (0–70), where higher scores indicate greater wellbeing. Keyes (2014) criteria for scoring this measure enabled us to categorise participants’ wellbeing as languishing (low wellbeing, characterised by emptiness, despair, and stagnation), moderate, or flourishing (high wellbeing, characterised by high levels of positive emotion, functioning well psychologically and socially). The MHC-SF has demonstrated moderate test–retest reliability and good convergent and discriminant validity with existing measures (Lamers et al., 2011).

### Post-assessment interviews

Individual, semi-structured interviews with seven of the 16 participants probed the feasibility of using the app. Prepared questions asked about the acceptability and clarity of the MuPsych questions and the accuracy of the data (e.g., how typical it was of their usual music listening habits) and we solicited any further practical feedback (e.g., about technical issues such as app installation, music listening app integration, and the process of answering questions). Interviews lasted between 10–25 min and were audio-recorded within Zoom.

## Data analysis

SPSS (version 27) was used to calculate descriptive statistics. Considering the items for wellbeing, choice and success outcomes were scored on seven-point scales ranging from-3 to +3, a score of zero represents a neutral outcome, a more positive score represents a better outcome, and a negative score represents a worse outcome. Linear mixed effects models were fitted to consider differences in listening episode wellbeing, choice and success by baseline wellbeing categorisation (namely, languishing, moderate, flourishing). These models consisted of the time point (5 or 20 min from the start of the listening episode) and baseline wellbeing categorisation as fixed effects with full interactions; and random effects of episode were nested within participant. These fitted models were analysed both cross-sectionally and longitudinally. The models were fitted using the lmer function from the lme4 package (Bates et al., 2015) in R (R Core Team, 2024), and contrasts were extracted from these models using the emmeans function from the emmeans package (Lenth, 2024). ggplot2 (Wickham, 2016) from the tidyverse meta-package (Wickham et al., 2019) was used for all figures, and tables were generated using functions from the kableExtra package (Zhu, 2024).

The seven interviews were transcribed and then listened to multiple times to identify comments made about topics relevant to the feasibility of the data collection method. Comments about similar topics were gathered together as a very basic form of inductive category assignment similar to content analysis (Mayring, 2015, p. 79), specifically, descriptive qualitative content analysis (Puppis, 2019). For example, all descriptions about frustrations were grouped together, as were comments about the frequency of questions, participants’ satisfaction with the available answers, their personal reasons for joining the study, as well as reflections on whether the data accurately represented their music listening habits.

## Results

### RQ1: practicality and engagement using a mobile ESM application

From the interviews, we learned that most participants were able to successfully download the app on their phone without assistance. One person, whose phone was purchased in China, was unable to get it working because it breached security limitations. Another person described being suspicious of the credibility of the app because of the security requests at set up (requesting access to all apps). Another person struggled to connect to their preferred listening app (YouTube) and found the app closed whenever they pressed ‘other’ when setting up MuPsych. Subsequently they needed to listen to music on a non-preferred tool which influenced their music listening and quality of their data. Many people described accessing music via multiple apps which is not able to be captured by MuPsych, where only one listening technology can be selected.

As a sample, participants reported on 263 episodes of listening to music. Individuals completed between 2 and 44 entries (*M* = 17; *Mdn* = 13). The dataset consists of 263 entries a 0-min, 163 entries at 5-min, and 47 entries at 20-min. Therefore, the use of an event sampling frame was suitable for the study aim; however, the frequency of prompts was a discussion point for many in the interviews, where people described being prompted more often than expected. This is a known influencer in ESM studies, with fundamental recommendations suggesting that it is important to avoid interrupting participants when they do not have time to respond (Van Berkel and Kostakos, 2021).

- “Too often for me because I forget about the surveys and then suddenly there would be a notification that pops up and disrupts. I tried to get used to it and it got a bit better. It bought a lot of annoyance at first”.

Some participants described how they used the app’s user controls to adjust the length of time between 0-min survey alerts (i.e., how frequently they were asked to complete surveys), but others expressed more confusion. One person usually listened for short bursts and had to adjust their listening style to listen for longer to be able to contribute to the study. They only realised this when listening for longer than usual in the shower.

- “I was in there (the shower) for 10–20 min and when I came out, I could see there was a notification and that made me realise that it needed to be a longer time”.

Another person naturally listened to music for longer but found that the app would start again when she moved from her car to walking because it re-triggered the surveys to begin again as a separate listening episode. They also described how the easiest time to use the app was at home because it was not stressful to answer the questions.

- “The only times I found it hard to do was when I was commuting, and it would pop up and I would get stressed that I would lose it. It was easier when I was cleaning the house or something it was easy to answer the question quickly”.

When asked about the suitability of the questions for gathering information about their intentions for music listening, several participants reported difficulty in choosing a goal. For some people it was because they had more than one goal, or because it was a background activity, rather than something with intention.

- “It was hard for me to choose just one, there was usually a lot of things I am doing”.
- “This one always tripped me up. I did not always know why I was listening – it was just because”.
- “It expects the music to have a bigger impact than it actually does sometimes. Sometimes it’s just there and it does not make me feel better or worse. For me, it was to have background in the shower that was the main reason. Just for something to do”.
- “Sometimes it is to motivate me to do housework – which is goal orientated. And then for working out it can be good. And then sometimes to pick up mood. But it needed to have the nothing option. It’s not always relaxing or distracted – it’s just to listen to it”.

### RQ2: efficacy of mapping music listening outcomes to wellbeing

**Figure 1:**
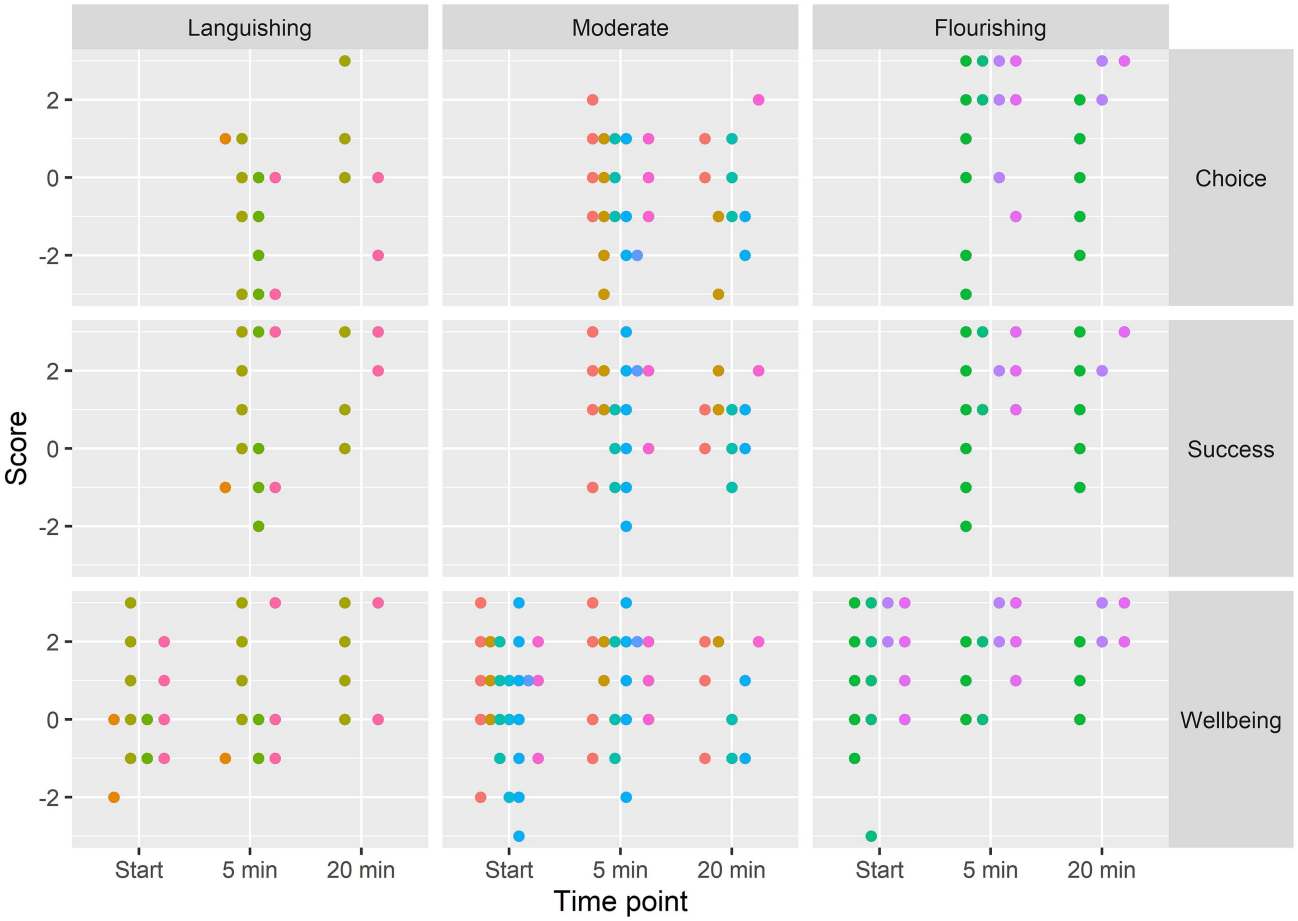
Raw scores grouped by outcome, time point and baseline wellbeing category. Scores at the same time point have been horizontally jittered so that they do not overlap, and different colours have been used to distinguish observations from different participants.

The first point of analysis was to examine patterns of music listening by baseline wellbeing level (using Keyes’ languishing, moderate, and flourishing categorisation). Figure 1 presents the raw scores for episode music choice, success of goal attainment, and current self-rated wellbeing by baseline wellbeing categorisation (number of participants per category [one participant did not complete the MHC-SF]: languishing (4), flourishing (4) and moderate) (neither languishing nor flourishing) (7) and timepoint (different colours have been used to distinguish data from different participants). The distribution of listening episodes for wellbeing and success were mostly positive, with most of the data points falling between 0 and 2 on the scale. The flourishing group tended to report more positive scores overall compared to the other two groups.

**Figure 2:**
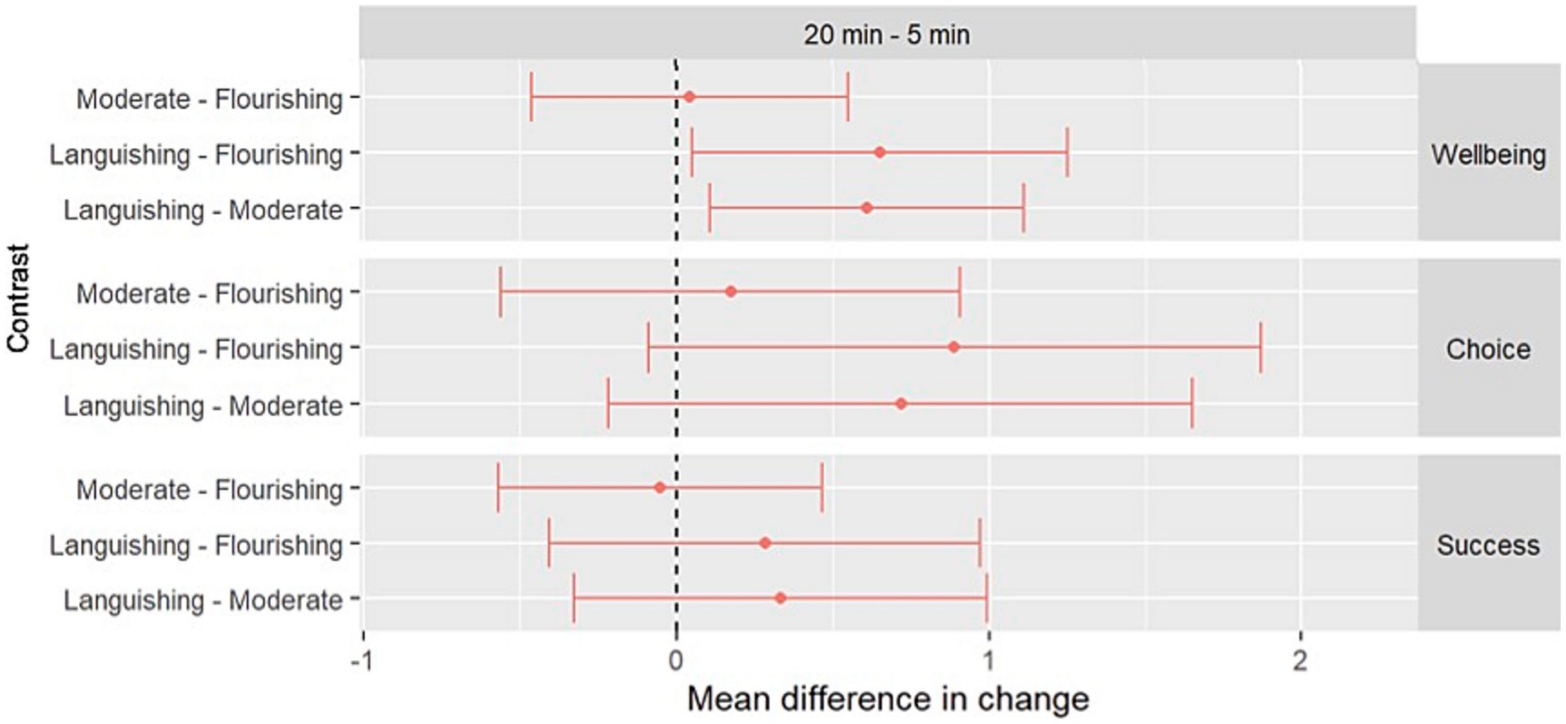
Estimated differences in changes in mean score between baseline wellbeing categorisation pairs by outcome.

Figure 2 illustrates the differences in changes between baseline wellbeing categorisation pairs from Linear Mixed Effects models. The languishing group had significantly larger changes in wellbeing scores from 5 to 20 min than the other two groups (see Table 1 for the statistical details pertaining to Figure 2; see Supplementary Table S1 for estimated mean changes in each outcome between 5 and 20 min by baseline wellbeing group). Figure 2 (see also Table 1) also highlights the lack of difference in change in wellbeing and success between the moderate and flourishing groups.

**Table 1:** Estimated differences changes in mean scores for each outcome between wellbeing categories.

			Difference in change					
Outcome	Period	Contrast	Estimate	*SE*	*df*	95% CI	*t*-ratio	*p*-value
Wellbeing	20 min – 5 min	L - M	0.61	0.25	59	0.11, 1.11	2.43	0.018
		L - F	0.65	0.30	56	0.05, 1.25	2.17	0.034
		M - F	0.04	0.25	58	−0.46, 0.55	0.16	0.871
Choice	20 min – 5 min	L - M	0.72	0.47	63	−0.22, 1.65	1.53	0.131
		L - F	0.89	0.49	60	−0.09, 1.87	1.81	0.074
		M - F	0.17	0.37	62	−0.56, 0.91	0.47	0.640
Success	20 min – 5 min	L - M	0.33	0.33	56	−0.33, 1.00	1.01	0.315
		L - F	0.28	0.34	55	−0.41, 0.97	0.82	0.416
		M - F	−0.05	0.26	55	−0.57, 0.47	−0.20	0.841

L = languishing, M = moderate, F = flourishing.

The categorisation of each participant was not known to the interviewer and the data collected cannot be interpreted with regards to flourishing or languishing. However, it was useful in understanding whether the data was accurately capturing the connection between music listening, goal achievement and wellbeing.

One issue noted by interviewees was the way the app listed the name of the song that had been playing when they answered the first questions (0 min) when they were being asked about goal achievement. This was supposed to link to the focus of the research on understanding whether people’s intentions were satisfied by their song selections. However, many people felt either confused or frustrated by this (especially at 20 min), because they had listened to several songs and their mood or goals may have changed. Some even described trying to write down the songs that had been played in between so they were better able to answer the question.

- “Half the time, the song did not come up. But when it did come up, it wasn’t the song I was currently listening to – it might have been a song that was from earlier questions, and then it would change. So, your mood can change in between… It never functioned as a helpful reminder. You could probably do it in the second lot of questions, but the third lot of questions were too far away, and it was asking about the first listening”.

It also seemed that the data may have been skewed by the motivations of some participants who did the study through a university research participation program (i.e., for the course credit) but who did not have strong music listening habits. They described adjusting their lifestyle to complete the study and contribute data; however, as a result, this data did not reflect their usual listening behavior.

- “I tried to be as authentic as possible but knowing that I was distracting myself made me aware of my mood and my feelings a lot more, but I did try and stick to how I was feeling at the time. But I was made more aware of what I was doing.”
- “I tried to sort out new music because I was going to be listening more for 2 weeks and did not want to listen to the same stuff all the time.”
- “I also listened differently because it was the requirement of the experiment. So, it wasn’t the same as the way I would use music usually.”

In addition, some described how participation in the study altered their behavior because they became more aware of how their choices could influence their wellbeing. This realisation also led to adjustments.

- “I feel more conscious of my intentions when I turn on the music. Previously I just turned on the music and did not think. And now I really think about why I’m using this music.”
- “I was a bit more intentional in experimenting with music after this. I tried playing songs that were a bit more uplifting along the way and I felt a bit better too.”
- “It bought attention to my habits because I would have to go through and read the list of why you are listening and that made me think about that, so then I would actually make myself get back to it.”

## Discussion

Our analysis revealed that categorising wellbeing into languishing, moderate and flourishing groups can be useful for answering questions about the relationship between music listening intentionality, and wellbeing. Even with a small sample, changes were observed between categories. The largest positive change in episode wellbeing was evident for the languishing group, which is something that deserves additional research attention. However, it is important to consider challenges to ecological validity when including a psychometric tool at baseline that asks participants to reflect on the past four weeks to determine their wellbeing state alongside ESM data focused on current state. Moreover, using a single item to probe level of wellbeing at each music listening episode was adequate; therefore, we recommend this approach be taken for future data collection as to not unduly lengthen the time it takes to complete baseline testing.

One noted challenge for studying people’s music use is that the increased awareness of behavior results in behavior change, which was evidenced in this study. Bourdieu’s theories about the pre-reflexive “habitus” (Bourdieu and Nice, 2013) have been used to explain how the unintended consequence of engaging in discourse about a topic is to change the informants understanding of it, leading to biased data. This was evidenced in some of the comments made by interviewees in the study (e.g., consciously listening to music for longer periods and meta-cognitive consideration of listening goals).

The interview data also revealed the challenges of collecting event-triggered data from people who were not music listening enthusiasts, since they produced minimal data (or had to adjust their music listening habits to meet the requirements of the study). Future studies should recruit people who are enthusiastic music listeners, rather than focusing on anyone who would be interested in doing a study on music listening.

Similarly, it is worth considering how to collect more appropriate data about people’s listening practices by sampling at random times with a 5-and 20-min follow up rather than only using the event-based sampling approach. Such an approach might capture contextual information as well as frequency and duration, inclusive of gathering data from people who have high and long periods of listening.

There was also a need to be able to capture listening experiences for people whose listening was not goal focused. A simple adjustment would be to include an additional response option (e.g., of “no purpose”). Similarly, it will be important for future research to ask participations to restate their goals at each time point since listening unfolds and it would be interesting to see whether their goals are stable or evolving.

Future studies might incorporate an initial meeting with participants to educate them about the app set-up and question sequencing (Fritz et al., 2024). This would serve the purpose of both checking the suitability of participants for the study and ensuring that frustrations and confusions could be addressed.

We found value in conducting interviews after data collection. ESM data is complex and being able to check the degree to which people had answered honestly, understood the questions, and altered their behavior in response clarified how much to rely on the quantitative data. This combination of rich *in situ* data and insights from the participants is necessary to triangulate such a complex data set.

## Data Availability

The datasets presented in this article are not readily available because ethics approval for this project does not permit sharing the collected data. Requests to access the datasets should be directed to k.mcferran@unimelb.edu.au.
