# Supplementary material for: Exploring the feasibility of collecting music and wellbeing data to examine intentional listening using a mobile-ESM application
Source: Front Psychol. 2025 Feb 28;16:1505929. doi: 10.3389/fpsyg.2025.1505929 (PMC11906668; doi:10.3389/fpsyg.2025.1505929)
Supplement: Supplementary file 1 [file Table_1.DOCX]

Supplementary Material

# Supplementary Table

Supplementary Table 1 shows the changes in each outcome between five and 20 minutes for each baseline wellbeing category. A significant improvement in wellbeing is evident for the languishing group, but not for the moderate or flourishing group who are roughly the same at 5 and 20 minutes.

## Supplementary Table 1

*Estimated Mean Changes in Each Outcome Between 5 minutes and 20 minutes by Baseline Wellbeing Category*

|  |  | Mean change (20 min – 5 min) | | | | | |
| --- | --- | --- | --- | --- | --- | --- | --- |
| Group | Outcome | Estimate | *SE* | *df* | 95% CI | *t*-ratio | *p*-value |
| Languishing | Wellbeing | 0.52 | 0.21 | 56 | 0.10, 0.94 | 2.47 | 0.017 |
|  | Choice | 0.64 | 0.40 | 62 | -0.17, 1.44 | 1.59 | 0.118 |
|  | Success | 0.27 | 0.28 | 55 | -0.30, 0.83 | 0.94 | 0.352 |
| Moderate | Wellbeing | -0.09 | 0.14 | 64 | -0.36, 0.18 | -0.67 | 0.508 |
|  | Choice | -0.08 | 0.24 | 67 | -0.55, 0.40 | -0.33 | 0.746 |
|  | Success | -0.07 | 0.17 | 58 | -0.41, 0.27 | -0.41 | 0.685 |
| Flourishing | Wellbeing | -0.13 | 0.21 | 55 | -0.56, 0.29 | -0.62 | 0.538 |
|  | Choice | -0.25 | 0.28 | 58 | -0.81, 0.31 | -0.90 | 0.374 |
|  | Success | -0.02 | 0.20 | 53 | -0.41, 0.38 | -0.09 | 0.932 |
